# Biological controls for standardization and interpretation of adaptive immune receptor repertoire profiling

**DOI:** 10.7554/eLife.66274

**Published:** 2021-05-26

**Authors:** Johannes Trück, Anne Eugster, Pierre Barennes, Christopher M Tipton, Eline T Luning Prak, Davide Bagnara, Cinque Soto, Jacob S Sherkow, Aimee S Payne, Marie-Paule Lefranc, Andrew Farmer, The AIRR Community, Magnolia Bostick, Encarnita Mariotti-Ferrandiz

**Affiliations:** 1 University Children’s Hospital and the Children’s Research Center, University of ZurichZurichSwitzerland; 2 CRTD Center for Regenerative Therapies Dresden, Faculty of Medicine, Technische Universität DresdenDresdenGermany; 3 Sorbonne Université U959, Immunology-Immunopathology-Immunotherapy (i3)ParisFrance; 4 AP-HP Hôpital Pitié-Salpêtrière, Biotherapy (CIC-BTi)ParisFrance; 5 Lowance Center for Human Immunology, Emory University School of MedicineAtlantaUnited States; 6 Perelman School of Medicine, University of PennsylvaniaPhiladelphiaUnited States; 7 University of Genoa, Department of Experimental MedicineGenoaItaly; 8 The Vanderbilt Vaccine Center, Vanderbilt University Medical CenterNashvilleUnited States; 9 Department of Pediatrics, Vanderbilt University Medical CenterNashvilleUnited States; 10 College of Law, University of IllinoisChampaignUnited States; 11 Center for Advanced Studies in Biomedical Innovation Law, University of Copenhagen Faculty of LawCopenhagenDenmark; 12 Carl R. Woese Institute for Genomic Biology, University of IllinoisUrbana, IllinoisUnited States; 13 IMGT, The International ImMunoGeneTics Information System (IMGT), Laboratoire d'ImmunoGénétique Moléculaire (LIGM), Institut de Génétique Humaine (IGH), CNRS, University of MontpellierMontpellierFrance; 14 Laboratoire d'ImmunoGénétique Moléculaire (LIGM) CNRS, University of MontpellierMontpellierFrance; 15 Institut de Génétique Humaine (IGH), CNRS, University of MontpellierMontpellierFrance; 16 Takara Bio USA, Inc.Mountain ViewUnited States; United States; Institute of Industrial Science, The University of TokyoJapan

**Keywords:** T-cell Receptor (TCR), B-cell Receptor (BCR), next generation sequencing (NGS), immunoglobulin, antibody, IG, TR

## Abstract

Use of adaptive immune receptor repertoire sequencing (AIRR-seq) has become widespread, providing new insights into the immune system with potential broad clinical and diagnostic applications. However, like many high-throughput technologies, it comes with several problems, and the AIRR Community was established to understand and help solve them. We, the AIRR Community’s Biological Resources Working Group, have surveyed scientists about the need for standards and controls in generating and annotating AIRR-seq data. Here, we review the current status of AIRR-seq, provide the results of our survey, and based on them, offer recommendations for developing AIRR-seq standards and controls, including future work.

## Introduction

Immunoglobulin chains (IG) and T-cell receptor chains (TR) are generated by DNA recombination, a process of somatic rearrangement of variable (V), diversity (D), and joining (J) genes (Tonegawa, 1983). The diversity of the resulting rearranged genes (referred as V-J and V-D-J) is very high, due to not only the combination of different germline V, D, and J genes, but also to the deletion and addition of templated (P) nucleotides and the addition of non-templated (N) nucleotides at the junctions between the rearranged genes, and somatic hypermutation of expressed IG (Papavasiliou and Schatz, 2002; Lefranc and Lefranc, 2020). The total number of potential expressed rearranged IG and TR sequences in an individual is referred to as the adaptive immune receptor repertoire (AIRR). The adaptive immune repertoire is very diverse in a healthy individual, with the theoretically possible number of clonotypes reaching more than 10^19^ different TR (Bradley and Thomas, 2019) and 10^11^ IG (Glanville et al., 2009), far exceeds the number of B and T cells in a given individual (Davis and Bjorkman, 1988; Freeman et al., 2009; Elhanati et al., 2015). Thanks to next-generation sequencing (NGS), the AIRR can be sampled with sufficient depth for some of its complexity to be studied (Weinstein et al., 2009; Six et al., 2013). AIRR sequencing (AIRR-seq) provides insights into the immune status of an individual at steady-state or in altered conditions such as malignancy, autoimmune disease, immunodeficiency, infectious disease, or vaccination, and allows comparison of B- and T-cell populations between individuals and time points (Benichou et al., 2012; Kirsch et al., 2015; Dziubianau et al., 2013; Hou et al., 2016; Ghraichy et al., 2018). AIRR-seq permits the description and quantification of global diversity and characteristics of AIRR, the identification of clonal expansions, the tracking of particular clonotypes, and the prediction of their specificities (Miho et al., 2018; Zvyagin et al., 2020; Sidhom et al., 2018; Glanville et al., 2017; Huang et al., 2020; Jokinen et al., 2021; Akbar et al., 2021; Hayashi et al., 2021) as well as the antibody selection through phage display (Rouet et al., 2018; Ravn et al., 2013), thereby providing opportunities for new biomarker identification (Gittelman, 2021; Dines, 2020), therapeutic antibody discovery (Akbar et al., 2021; Richardson et al., 2021), CAR-T cell bioengineering (Sheih et al., 2020), vaccine development, cancer diagnostics and treatment (Linette et al., 2019; Zhang et al., 2018; Lu et al., 2018), including neoantigen discovery (Chiou et al., 2021; Richters et al., 2019) and immune intervention monitoring in diverse pathologies, such as stem cell transplantation (Robinson, 2015; Fink, 2019; Jiang et al., 2019; Jacobsen et al., 2017; Theil, 2017; Link-Rachner et al., 2019; Rubelt et al., 2017; Parola et al., 2018; Georgiou et al., 2014; Arnaout et al., 2021; Anand et al., 2021).

NGS-based approaches and methods have multiplied, now including high-throughput bulk sequencing of IG or TR starting from genomic DNA (gDNA) or mRNA (as cDNA), which typically provides information on one receptor chain only, and more recently to the sequencing of the two IG or TR chains expressed in a single cell, which provides information on the antigen-specific receptor. These approaches are increasingly applied, mostly to human AIRRs, but also to study AIRRs from other organisms (Chaudhary and Wesemann, 2018; Minervina et al., 2019). Molecular protocols to amplify IG or TR chains typically rely on Polymerase Chain Reaction (PCR), such as multiplex-PCR or RACE-PCR (Robins et al., 2009; Wang et al., 2010; DeKosky et al., 2013; Eugster et al., 2013; Heather et al., 2015; Mamedov et al., 2013).

To obtain reliable and comparable AIRR-seq data, the methods for performing AIRR-seq need to fulfill a number of requirements. First, the data generated by AIRR-seq must reflect the composition and diversity of the ‘real cellular repertoire.’ The cell subset(s), the cell sample size, and the sequencing depth used in an AIRR-seq experiment all influence the downstream data and should therefore be carefully adapted to the experimental question (Rosenfeld et al., 2018). PCR amplification of AIRR-seq libraries can introduce bias by preferentially targeting certain genes, by missing certain alleles (in the case of multiplex PCR with primers anchored in the V or J genes), and by overamplifying targets of certain genes and length (in multiplex PCR and RACE-PCR) (Calis and Rosenberg, 2014; Alamyar et al., 2012; Gadala-Maria et al., 2015; Primi et al., 1986; Barennes et al., 2021). All these parameters will influence how accurately AIRR profiling reflects the true abundance and diversity of clones in the immune repertoire. Second, AIRR V and J genes (and constant (C) genes for IG) must be identified in an unambiguous and unbiased manner as knowledge of the complementarity determining region 3 (CDR3; the site of V, (D), and J recombination in an IG or TR, and hence its greatest sequence diversity), but also of the CDR1 and CDR2 (and that of the C gene in IG) is critical to assess the physicochemical constraints that define specificity, affinity, and function of the TR or the IG (Li et al., 2013; Rossjohn et al., 2015). Third, AIRR-seq data should be as free from sequencing error as possible. The CDR3, key for assigning a sequence to a clonotype, is by definition unknown, and in the case of IG sequences it is important to be able to distinguish *bona fide* somatic hypermutation from artifactual mutations introduced by PCR or by sequencing errors all along the rearranged molecule, as the latter can generate falsely elevated inter- and intra-clonal variation. Further complicating the matter is the fact that the germline V genes from the same subgroup (e.g., IGHV4-31 vs. IGHV4-30-4 for IG) and V alleles or polymorphic variants of a given gene (e.g., TRAV14/DV4 for TR) may have very few nucleotide differences (Lefranc, 2014; Lefranc and Lefranc, 2001). Therefore, distinguishing errors from true biological variants can be a major challenge. Finally, samples for AIRR-seq should be free from cross-sample contamination, to which AIRR-seq experiments are prone as multiple samples are often processed in parallel and sequenced on a single lane of a sequencing run.

In the past decade, multiple molecular biology protocols and approaches have been developed by academic and industrial investigators, rendering comparisons among studies difficult. Moreover, experimental and analytical protocols are highly complex and therefore prone to intra- and inter-experimental variability (Barennes et al., 2021; Liu et al., 2016; Rosati et al., 2017; Bashford-Rogers et al., 2014). AIRR-seq can be performed either on gDNA or cDNA from multiple T/B cell populations (bulk sequencing) or on individual cells (single-cell sequencing). The starting material and sequencing method used depend on the application, as each has advantages and disadvantages (Table 1). The availability of commercial kits can be helpful, since they are produced following standards and rigorous quality control, thus offering standardized reagents and protocols across laboratories. Currently, available commercial kits include gDNA-based methods (e.g., Adaptive Biotechnologies, iRepertoire) as well as mRNA-based methods that use cDNA (e.g., Illumina, Takara Bio, iRepertoire) for bulk sequencing. Mainly mRNA-based commercial methods (e.g., 10X Genomics, Takara Bio, HiFiBio) are used for single-cell analysis, which can provide sequences for full receptors or antibodies. This is an important consideration as the determination of the isotype for IG requires a full or partial sequence of the constant region. Single-cell approaches are further helping in the detection of clonotypes because they provide both paired chains and potentially allow full-length cDNA sequencing (Stubbington et al., 2017). The fidelity of sequence is an additional factor to consider. Unique molecular identifiers (UMI) consisting of random stretches of 8–12 nucleotides are incorporated into oligonucleotides that are used to generate cDNA from mRNA, such that statistically each cDNA molecule contains a unique sequence. Analysis of sequences that share the same UMI is used to generate a consensus sequence, greatly reducing sequencing errors (Shugay et al., 2014). In contrast, multiplex PCR approaches can be associated with artifacts arising from primer competition or off-target primer binding. Although this favors RACE-PCR when considering mRNA-based methods, gDNA-based multiplex PCR may offer higher fidelity since it does not rely on reverse transcription (reverse transcriptase enzymes have higher error rates than DNA polymerases [Ellefson et al., 2016; Holland et al., 1982]). Finally, cost may influence the choice of a particular protocol. There are many factors that contribute to the cost of AIRR-seq data generation. For example, the cost of sequencing, the sequencing depth, and the number of cells analyzed per sample are variable; also, the choice between commercial kits and ‘homebrew’ methods will influence costs. In general, gDNA analysis is the most cost-effective, because it requires the lowest sequencing depth with the largest representation of cells per sample, whereas single-cell analysis is on the opposite end of the scale, with bulk cDNA sequencing in the middle.

**Table 1.** Current AIRR-seq methods and their typical use(s). Bulk gDNA, bulk cDNA, and single-cell cDNA-based sequencing methods are compared with respect to their general features, uses, methods, and potential issues. Each is ranked using a semi-quantitative scale (from ‘+++” for best to ‘-” for worst or non-existent).

		Bulk gDNA sequencing	Bulk cDNA sequencing	Single-cell cDNA sequencing
General Features	PCR method	Multiplex	Multiplex and 5' RACE	Multiplex and 5' RACE
	Cell number	10^2^–10^6^	10^2^–10^6^	10^2^–10^3^
	Sample throughput	Low-high	Low-moderate	Low
	Length of receptor sequences	100–600 bp	150–600 bp	700–800 bp
	Availability of commercial kits and service providers	++	+++	+
				
Uses	Gene usage	++	++	+
	CDR3 length and properties	++	++	+
	Somatic hypermutation (for IG)	++	++	+
	Repertoire diversity	++	++	+/-
	Clonal expansion	+++	++	+
	Clonal evolution	++	+++	++
	Tracking of clonotypes	+++	++	+
	Clinical use (e.g., MRD detection)	++	+/-	-
	Unbiased detection of unproductive rearrangements	++	-	-
	Inference of germline	++	+	+/-
	Determination of constant gene	-	++	+
	Structural annotation	+/-	++	+
	Linkage of both antigen receptor chains	+/-	+/-	++
	Direct combination of AIRR-seq with single-cell immunophenotype (e.g., transcriptome or cell surface protein expression)	-	-	++
	Characterization of clonotype full antigen receptor/Functional testing	-	+/-	++
	Rare clonotype detection	++	++	+/-
				
Methods	Simplicity of workflow (library preparation)	+++	++	+
	Cost for library preparation commercial kits (per sample)	Low	Moderate	High
	Fidelity in sequences	Moderate	High	High
	Molecular barcoding (correcting PCR/sequencing error)	+/-	++	++
				
Potential Issues	V-gene amplification bias	++	+	+/-
	V-gene annotation issues	++	+	+
	PCR and sequencing error	++	+	+/-
	Difficulty with translation of copy number to cells	+/-	++	+/-
	Degradation of template	+	++	++

bp = base pairs; CDR3 = complementarity determining region 3; MRD = minimal residual disease; RACE = rapid amplification of cDNA ends; V = variable.

Several considerations should be taken into account when designing and planning an AIRR-seq experiment. In addition to the large number of different methods and protocols, other factors including budgetary constraints, timelines, sample types, and processing are also important. Given the diversity of AIRR-seq workflows, comparisons between different data sets are challenging or even impossible. Standards and controls are needed for optimal AIRR-seq data harmonization, interpretation, and sharing (Rubelt et al., 2017; Breden et al., 2017; Vander Heiden et al., 2018). This need led to the formation in 2015 of a grassroots community of scientists and other interested parties, known as the AIRR Community (https://www.antibodysociety.org/the-airr-community/). The objective of the Biological Resources Working Group (WG) within the AIRR Community is to coordinate the assessment and development of AIRR-seq controls, ultimately providing the scientific community with controls and standards for the generation, harmonization, and rigorous comparison and interpretation of AIRR-seq data. In order to recommend biological standards that are needed and to prioritize their development, a written survey was developed asking participants about the use of and need for controls for AIRR-seq experiments. The survey gathered information on participants’ research interests, sample types, sequencing methodologies, currently available controls, and desired controls. In addition to the survey results, different AIRR-seq methods and controls were also gleaned from the literature, and finally, the WG also invited scientists with unpublished research on controls for their input. These three sources of data were then used to provide a comprehensive overview of sequencing methods, technical issues, current standards, and potential priorities for the development of future standards. Here we describe the progress of the Biological Resources WG of the AIRR Community, its information collection and proposed strategies to define, develop, and use AIRR-seq standards.

### Biological controls in AIRR-seq experiments

#### AIRR Community survey: Overview and respondent demographics

**Figure 1.**
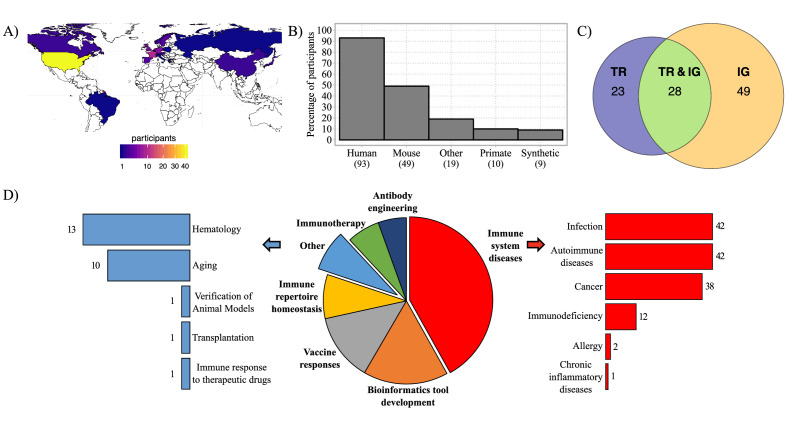
Geographic distribution of survey participants and their AIRR-seq research interests. (**A**) Map with geographic distribution of survey participants. (**B**) Histogram showing the principal studied organisms among the participants. The ‘Other’ category includes rat, ferret, rabbit, goat, pig, canine, bovis, cattle, chicken, fish, teleost, salmon, zebrafish, other fish species, transgenic animals. (**C**) Venn diagram representing the percentage of participants according to their interest in AIRR template type. (**D**) Pie-chart representing the distribution of survey participants according to their research interest(s). Immune system diseases and other categories are described in more detail in the bar plots (right and left). Numbers of respondents for each category are shown next to the bars.

To address the use, needs, and requirements for AIRR-seq controls and standards, we designed and disseminated a questionnaire to researchers in the AIRR Community, as well as to users of IMGT, the international ImMunoGeneTics information system (http://www.imgt.org) (Lefranc, 2014). The questionnaire was composed of 4 sections and included 28 questions, with tick-box predefined answers and free-text options allowing for participants’ personalized answers (**Supplementary material**). After 6 months, 105 responses were recorded, including one incomplete response from a participant who neither produces nor analyzes AIRR-seq data. Three respondents participated twice, with consistent answers and same name and contact information, therefore only one completed form was considered for each. Answers from 101 remaining participants originated mainly from North America and Europe (Figure 1A) and were further analyzed. At the time of the survey, 96% of respondents were involved in AIRR-seq studies and 4% had plans to perform AIRR-seq studies in the future. Of the respondents, 92% were engaged in human studies, 48% in mouse studies, and 38% in the use of AIRR-seq to study other species or synthetic molecules (e.g., from phage-displayed antibody libraries; Figure 1B). Approximately half of the respondents focused exclusively on IG while a quarter each studied TR and IG or TR alone (Figure 1C). Several respondents were interested in many different topics (Figure 1D and Figure 1—figure supplement 1), with their fields of interest dominated by ‘immune system diseases’ including infection, autoimmune disease, and cancer. Furthermore, the survey results clearly indicate an interest among the majority of respondents in developing bioinformatic tools for the analysis of AIRR-seq data, followed by major interests in other research areas such as vaccinology, immune repertoire homeostasis, immunotherapy, antibody engineering, hematology, and aging (Figure 1D). In addition, respondents with bioinformatic skills tended to be those who had broader research interests (Figure 1—figure supplement 1). Finally, 89/101 of survey respondents indicated an interest in using AIRR-seq to either track clonotypes over time or across samples, whereas 88/101 were interested in identifying highly expanded clonotypes, 87/101 on analyzing diversity, and 83/101 on studying clonal selection. In conclusion, participants in this survey came from diverse backgrounds, had wet and dry bench expertise, and had a breadth of research interests covering different aspects of AIRR-seq studies.

**Figure 1—figure supplement 1.**
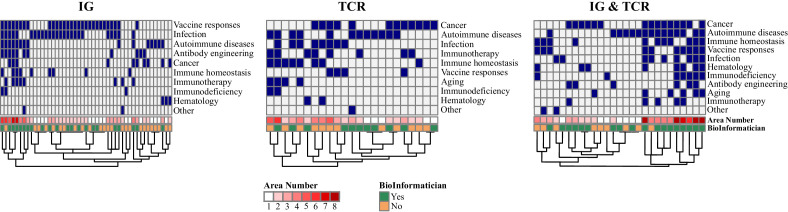
Heatmaps of the areas of study depending on the interest. Each column is a participant group and the colors represent the area studied (blue: area studied, white: area not studied). For each heatmap, areas are ordered by decreasing frequency depending on the number of participants in each group. Information about the number of areas per participant and bioinformatic skills are added below (Chi2: p=0.0064).

### Survey results on sequencing methodologies used

**Figure 2.**
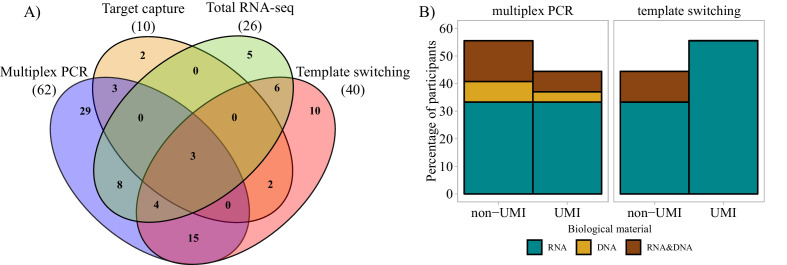
Molecular approaches used in bulk sequencing. (**A**) Venn diagram representing the most important molecular approaches used and their usage and sharing among participants. Numbers of respondents in each of the four main categories are shown in parentheses. (**B**) Bar plots representing biological material used and molecular barcoding proportion for the two major molecular biology approaches (multiplex PCR and template switching). Only the answers of respondents who used one technology exclusively are shown (Multiplex PCR: n = 29; Template switching: n = 10). UMI = unique molecular identifier.

Whereas 91% (92/101) questionnaire respondents commonly perform bulk sequencing experiments, 67% (68/101) combine bulk sequencing with the use of single-cell technologies. Only 8% of respondents focused exclusively on single-cell sequencing or phage display technologies only. With respect to the input biological material used in bulk sequencing, the majority of participants (83%) preferred to sequence long amplicons that covered the entire (or almost the entire) V-(D)-J region and part of the C region despite the associated higher sequencing cost per read and restrictions on the type of compatible sequencer. AIRR-seq researchers mainly used mRNA for cDNA sequencing (69%), while both mRNA and gDNA were used by 25%, and gDNA alone by 6% of respondents. Figure 2A shows that the majority of survey respondents performing bulk sequencing used multiplex PCR or the template-switching approach with a considerable number of AIRR-seq researchers using both methods. For those using either approach, mRNA was still the preferred starting material. In addition, UMIs were more commonly used with template switching than multiplex PCR approaches (Figure 2B). The association of UMIs with template switching methods is likely related to the template choice, since UMIs are not easy to use with gDNA-based templates, due to the incorporation of the UMI into the amplified products; nevertheless, two participants in the survey reported using UMIs with gDNA (Figure 2B).

**Figure 2—figure supplement 1.**
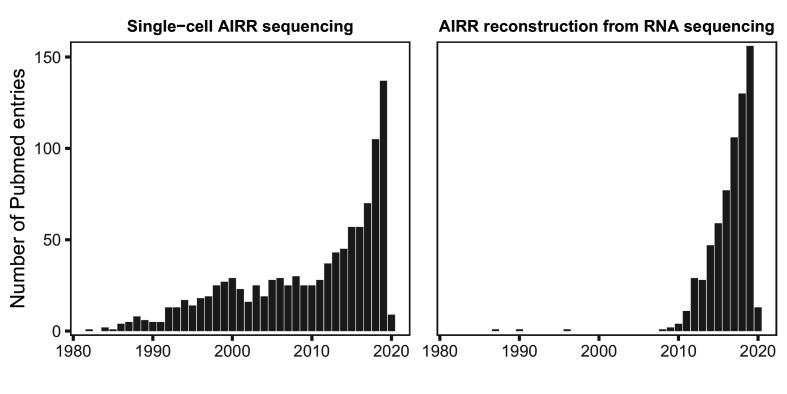
Yearly number of PubMed entries referring to single-cell AIRR sequencing (left panel) and bioinformatic AIRR reconstruction from RNA-seq studies (right panel) 1980–2020 (via https://www.ncbi.nlm.nih.gov/pubmed/, accessed on 16 January 2020). Search query (left panel): (‘single cell’[Title/Abstract] OR ‘10X’[Title/Abstract]) AND (‘VDJ’[Title/Abstract] OR ‘TCR’[Title/Abstract] OR ‘BCR’[Title/Abstract] OR ‘b cell receptor’[Title/Abstract] OR ‘t cell receptor’[Title/Abstract] OR ‘repertoire’[Title/Abstract]) Search query (right panel): (‘rna-seq’[Title/Abstract] OR ‘rna sequencing’[Title/Abstract]) AND (‘VDJ’[Title/Abstract] OR ‘TCR’[Title/Abstract] OR ‘BCR’[Title/Abstract] OR ‘b cell receptor’[Title/Abstract] OR ‘t cell receptor’[Title/Abstract] OR ‘repertoire’[Title/Abstract]).

Altogether, these initial results suggest that among survey respondents bulk sequencing on mRNA and gDNA are the most frequently used methods. However, the literature reflects increasing use of single-cell approaches, including the computational construction of IG or TR from RNA-sequencing (RNA-seq) libraries (Figure 2—figure supplement 1) and the combined use of target capture plus single-cell RNA-seq (e.g., 10X Genomics’ 5′ end kits, etc.).

To gain insight into which standards should be prioritized for development and sharing with the scientific community, we asked survey participants about the standards they currently use and about the types of standards they would like to use. Based on the results, we concentrate below on potential controls for bulk analysis, as this approach is being used by the majority of the respondents (91%) and because the development of standards for single-cell applications is somewhat distinct.

### Survey results on controls used and desired controls

**Figure 3.**
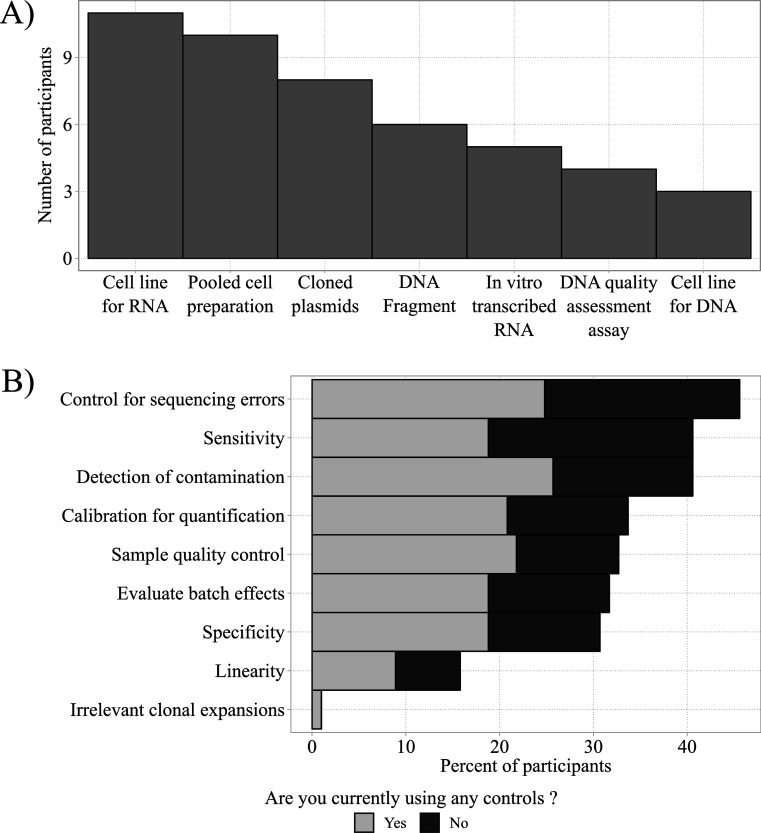
Homebrew controls and their desired applications. (**A**) Most frequently used homebrew controls (total n = 47). (**B**) Total frequencies of desired applications of homebrew controls for respondents currently using (gray bars; total n = 47) and currently not using controls (black bars; total n = 42).

Most respondents (88%) were interested in using standards or controls in AIRR-seq experiments. The 47 respondents who already use controls (n = 47) did so for protocol development (12/47 = 26%), everyday use (7/47 = 15%), or for both (15/47 = 32%), with the remaining (13/47 = 28%) not indicating their specific application. Commercial controls were used by 11, and homebrew controls were used by 23 participants, with 3 people using both and 16 not specifying their source. Figure 3A indicates that the most commonly used homebrew controls were cell lines or pooled-cell preparations. Respondents who did not use controls (black bars) were also asked how they might want to use controls. Figure 3B depicts the community survey responses to these questions.

In summary, the survey, as well as further discussions within the AIRR Biological Resources WG, identified major concerns arising from different steps in the AIRR-seq workflow (Table 2, left and middle columns). Additionally, based on these identified issues, the right column of Table 2 describes potential controls to address them. These controls are described in more detail in the subsequent sections of the manuscript.

**Table 2.** Concerns and expected errors introduced during AIRR-seq workflows and possible controls to detect them. A typical workflow consists of 5 steps: Sample collection > Extraction > Amplification > Sequencing > Analysis.

Concern	Mechanism(s)	Example of potential controls
Sequence errors	Enzyme errors (RT, DNA polymerase); Sequencing errors	UMIs for bioinformatic error correction; Spike-in controls with defined sequences to evaluate error rates
Sensitivity	Enzymatic inefficiencies (RT or PCR conditions/polymerase); Sample collection size (e.g., cell input number, purity); Sequencing depth	Spike-in controls (synthetic or cellular) at known concentrations
Specificity	Enzyme bias (RT, DNA polymerase); Analysis pipelines (annotation, error correction)	Spike-in controls with defined sequences to identify overall V/D/J gene amplification bias
Detection of contamination	Bench-level cross contamination (sample mixing or PCR contamination) or barcode jumping during sequencing	Unique spike-in (synthetic) for each sample; UDIs for sequencing barcode crosstalk
Sample quality control	Sample collection or nucleic acid purification	Identified by spectroscopy or agarose electrophoresis
Evaluate batch effects	Subtle differences introduced at all stages of the workflow	Spike-in controls (synthetic or cellular); Parallel biological (clonal or complex) sample
Linearity/accuracy of clonotype quantification	Enzymatic inefficiencies (RT or PCR conditions); Analytical error correction	Spike-in controls (synthetic or cellular) at known concentrations
Reproducibility/Batch effects	All stages	Spike-in controls (synthetic or cellular); Parallel biological (clonal or complex) sample; Comparison of replicate amplifications of the same sample; Comparison of sequences generated on the same sample in different sequencing runs
Data processing	Database/annotation limitations; filtering; error correction; collapsing/consensus algorithms	Spike-in controls (synthetic or cellular); Parallel biological (clonal or complex) sample

RT, reverse transcriptase; UMIs, unique molecular identifiers; UDIs, unique dual indices.

### Current concepts in the use of biological standards for RNA-seq experiments

To prioritize the development of community-wide standards for AIRR data, we turned to examples of community-wide standards in the NGS space. Several such standards have been developed and are actively used, for example, the External RNA Control Consortium (ERCC) spike-in controls and the Microarray Quality Control (MAQC) RNA standards. Both of these standards were generated through broad collaborations of stakeholders including the United States government, industry, and academic researchers (Reid and External RNA Controls Consortium, 2005; Su et al., 2014).

The ERCC spike-in controls were designed specifically to be used with RNA-seq experiments, for normalization of expression values during analysis. Two tubes with different compositions of the RNA sequences are commercially available from Thermo Fisher Scientific as Invitrogen ERCC RNA Spike-In Mix (https://perma.cc/WW9Z-D2NY) and ready for use with eukaryotic samples. Each tube contains 92 RNA species with each containing a predefined polyA tail with a different sequence, with their relative abundance covering a ~10^6^ fold range, and a limited range of GC content and lengths. The external RNA mixture can be used to normalize relative quantities of transcripts across samples within a single experiment or project, and to optimize protocols for reproducibility and accuracy. The ERCC standards are widely accepted by the transcriptome community, but do suffer from a few limitations: (i) the GC content and range of lengths are not broad - representing the average, but not all possible mRNA moieties; (ii) the polyA region is shorter than endogenous mRNA, and (iii) no splicing variants are included (Jiang et al., 2011). These limitations result from the typical compromise accompanying any process for generating useful (and well validated) controls in a timely fashion.

The MAQC RNA standards were not generated as NGS controls initially, but instead were developed by the U.S. Food and Drug Administration (FDA)-led, community-wide consortium for the purpose of validating microarrays, instruments, and analysis methods. The MAQC project consists of four phases, with the first two focusing on microarray methods and the second two on NGS methods (SEQC; Sequencing QC). Initially, differential gene expression levels of nearly 1000 genes between two human reference RNA samples (Human Brain RNA and Human Universal RNA) were assessed by qRT-PCR and microarrays; these highly characterized RNA samples were subsequently used to validate different microarray or NGS library preparation methods, instruments, and data analysis methods (Shi et al., 2006). Phase 3 of the MAQC project applied similar concepts to NGS platforms and the comparison of results obtained by microarray or RNA sequencing (Su et al., 2014). Phase 4 of this project is ongoing, with the goal of developing robust analysis protocols and providing quality control metrics.

In addition, UMIs and unique dual indices (UDIs) have been proposed in the RNA-seq field (https://perma.cc/AMB4-WC86) (Kircher et al., 2012) to control and correct for sequencing errors, as well as sequencing index crosstalk.

### Current practices for controls in AIRR-seq experiments

While the standards described above cannot be directly applied to AIRR-seq experimentation, they can serve as a blueprint for the development of standards. In Table 2 (right column), we highlight different possible approaches to address the concerns of the AIRR Community. These approaches closely resemble the general standards described above, using spike-in controls or well-characterized biological samples, including UMIs or UDIs. As described below, AIRR-seq researchers have already initiated some studies to address the use of such controls.

Several groups have recently developed synthetic standards for use with AIRR-seq samples of mouse and human origin, all based on common principles, and generally available for academic institutions via a material transfer agreement. These synthetic templates are either generated from plasmids (via in vitro transcription followed by RT-PCR) or directly produced as synthetic dsDNAs, with numbers of unique sequences produced ranging from dozens to over 1 million (Khan et al., 2016; Friedensohn et al., 2018; Carlson et al., 2013) (and unpublished work of J. Trück, University Children’s Hospital Zurich and C. Tipton, Emory University).

The first use of a synthetic repertoire was reported by Carlson et al., 2013. They developed synthetic DNA templates combining 14 and 4 different V and J genes of the TR gamma (TRG) locus, resulting in a total of 56 templates of equal length. All sequences contained three barcodes to unambiguously identify individual V-J combinations. In addition, universal primer sites that were identical in all synthetic templates were added on both ends. This approach allowed identification of amplification bias and optimization of primer concentrations as well as informing the computational correction of residual bias. Work on both murine and human IG repertoire was performed by Khan et al., 2016 and Friedensohn et al., 2018 using a similar strategy in the same research laboratory. There, a total of 16 murine and 85 human synthetic sequences were used to assess primer bias from multiplex PCR library generation. In combination with incorporation of UMIs into amplicons and an error correction analysis pipeline, this approach increased workflow fidelity and produced more accurate data. A very important element in the strategy used in this process was the integration of UMI during initial reverse transcription, resulting in labeling of each cDNA on a single molecule level. In contrast to the study by Carlson et al., synthetic sequences used in both studies from Khan et al. and Friedensohn et al. contained different CDR3 sequences and were used in different relative concentrations within the spike-in pool. This approach allowed to not only assess primer-dependent amplification bias but also the impact of variable input concentrations of synthetic sequences on their relative abundance following sequencing.

In principle, synthetic templates are designed such that each mimics an individual recombined V-(D)-J region and a partial C region while also containing universal priming sites, barcodes for unique template identification. The universal priming sites allow for unbiased quantification. Furthermore, comparison of amplification efficiency with the universal vs. targeted primers (the latter usually binding to the leader or V and J or C regions) may be used to correct for target-specific differences in amplification efficiency. Through amplification of synthetic templates alone, multiplex primer sets can be tuned to individual concentrations that will more accurately amplify known targets within repertoires or they can be used to eliminate primers that perform poorly altogether. Through the use of known templated sequences of known abundance (e.g., cell lines spiked into other cells), quantification and amplification efficiency can be calculated. Experience from early testing has identified certain limitations of this approach (see below). Some standards additionally harbor mutations deviating from the germline (unmutated) IGHV sequences; these can be used to model the efficiency of amplification of somatically mutated templates (Friedensohn et al., 2018). In practical terms, synthetic standards can be used during method development (primer optimization, alteration of methods to account for amplification bias, etc.), as spike-ins, or can be run as separate positive controls alongside the samples of interest. Synthetic templates can also serve as spike-in controls for concurrent quantification measurements of run-to-run variability, amplification and sequencing efficiency, as a positive control, or as a measurement of sample-to-sample contamination and/or index of mis-identification (using different synthetic library spike-ins for individual amplifications in a pooled sequencing run).

Although theoretically promising, the molecular design and bioinformatic analyses of synthetic sequences are challenging. Controls should mimic biological repertoires as closely as possible, and therefore are most effective when they contain a representative level of the biological diversity, which is tedious and expensive. In addition, they should also be distinguishable from other biological sequences so that even following nucleotide changes introduced through PCR or sequencing errors, such synthetic sequences can be unambiguously resolved from their biological counterparts. These challenges may explain the rare usage of spike-in controls except for initial method optimization or very specific applications.

Mixed-cell populations and cell lines can also be used as workflow controls, as has been documented recently by the Euroclonality Consortium (Knecht et al., 2019; Brüggemann et al., 2019). The first type of Euroclonality (Knecht et al., 2019) control monitors general primer and sequencing performance of a sequencing run (batch of samples) and consists of a poly-target control, comprised of gDNA isolated from healthy human thymus, tonsil, and peripheral blood mononuclear cells (PBMCs), the latter derived from apheresis donors (in a 1:1:1 ratio). This control is included in the workflow alongside experimental samples in a separate tube. By bioinformatic identification of the primer sequences and comparison to stored reference sequencing profiles from the sample control mix, unusual amplification patterns or batch effects can be identified. One advantage of such a cell mixture control is that it fully models the immune repertoire complexity of a bulk cell population and provides quality monitoring for every step of the process including amplification of the template and its sequencing. A second advantage is that this type of control is very easy to generate and is therefore accessible to many laboratories. A disadvantage of using this type of complex control is that one does not fully know the identity of all of the rearrangements in the sample, which can be problematic if there is sample or PCR contamination. A second disadvantage is that it is generated in a finite amount - and once used up, the process of validating the control must be repeated.

The second type of control used by the Euroclonality Consortium (Brüggemann et al., 2019) is designed to evaluate assay sensitivity and linearity within each library. This in-parallel control consists of a gDNA sample obtained from 59 human B/T lymphoid cell lines with a total of 46 well-defined rearrangements mixed together in different ratios and added to each processed sample. An advantage of using cell lines as in-parallel controls is that their gene rearrangements are defined and thus easily identified; theoretically allowing for the conversion of reads into cell numbers and permitting relative quantification of template abundance. In practice, however, the use of in-parallel amplification controls can be very challenging, and requires careful interpretation. For example, in samples with poor gDNA quality or low template abundance, the control templates may outcompete the test sample. The depth of sequencing and relative amounts of sample input can affect the measured abundance (Barennes et al., 2021; Chaara et al., 2018). An additional disadvantage is that cell lines do not model a fully diverse repertoire, only a fraction of V and J gene combinations are represented by the cell lines, and thus primer performance and bias, especially between samples, are not fully controlled.

### Discussion and future work

AIRR-seq experiments are becoming increasingly commonplace, in both the research and clinical settings. In contrast, the development of controls and best practices for assay validation, interpretation and standardization have lagged behind. Here, the members of the AIRR Community Biological Resources WG have summarized the current practice regarding the use of standards and controls among its members as well as among other international AIRR-seq experts and in the literature. We also have identified differences in the types of standards and controls that are used among users. Some of these differences depend on the sample type (fresh vs. fixed cells), the starting material (single cell vs. bulk), the template (mRNA vs. gDNA), as well as the quality and quantity of the relevant cells and templates. In addition, the selection of controls is influenced by the amplification method, with single cell and mRNA-based methods relying more heavily on cellular and molecular barcoding approaches, for example. Last, but certainly not least, the downstream application of the assay can profoundly influence the choice and prioritization of controls. In some cases, assays need to be sensitive and specific (e.g., a clinical grade assay that detects minimal residual disease) whereas in others quantitation (e.g., clonal size analysis for monitoring clonal expansions) or unbiased amplification (e.g., assessment of repertoire skewing during an immune response) may be more important.

All AIRR-seq assays can clearly benefit from rigorous controls. There is broad agreement that controls and standards are desirable, with over half of AIRR-seq survey respondents currently already using controls (mostly of a homebrew variety) in their experiments. Furthermore, whether individuals used controls or did not, they appeared to agree on the types of issues in analyzing and interpreting AIRR-seq data that would benefit from the use of controls - most importantly measuring and controlling for sample quality, assay sensitivity and specificity, and calibration for the quantification of clonal size. Also, with the progress regarding antigen-specificity inference using computational tools (Glanville et al., 2017; Huang et al., 2020; Jokinen et al., 2021; Akbar et al., 2021; Hayashi et al., 2021; Richardson et al., 2021; Galson et al., 2015; Shomuradova et al., 2020; Chronister et al., 2021; Sidhom et al., 2021; Pogorelyy et al., 2019; Dash et al., 2017) or more conventionally through technologically challenging using antigen-binding approaches, including single-cell (Johnson et al., 2020; Fuchs et al., 2019), having controls would be of major interest to ensure the accuracy of the TR or IG identified. It is unlikely that a single control can fulfill all of these needs across all methods and applications. There is at the moment also no obvious front-runner for a ‘gold standard’ that can be used to judge the adequacy of different types of controls.

Having identified a need, a diversity of methodologic approaches and the lack of a ‘gold standard’ for AIRR-seq controls, the AIRR Community Biological Resources WG is now coordinating the development of such controls. Although AIRR-seq researchers are aware of potential methodological problems, current solutions have not been systematically evaluated or compared. Based upon the current use of controls and needs identified by our survey respondents, the WG plans to focus on three forms of controls: one in-parallel (synthetic standards), one in-parallel and computational (UMIs), and one that is external (a complex cell mixture that is run in parallel to monitor amplification and sequencing run batch effects). To optimize these three types of standards, we first must determine how well they work. We therefore propose to carry out a multi-center analysis of three types of controls: (1) synthetic calibrators for bulk gDNA sequencing to measure clonal size and amplification bias; (2) UMIs for samples studied in parallel by bulk mRNA (with UMIs) and single cell sequencing (with cellular barcodes) for the analysis of amplification bias and sequencing error; and (3) a mixed cell population (either a human apheresis and/or pooled tissue product or murine spleen samples) for the evaluation of batch effects that compare between sequencing runs performed at the same site or between runs performed at different sites. These three types of controls can be run in parallel or in separate, dedicated experiments, allowing for greater participation of AIRR-seq investigators.

In order to perform these studies, the Biological Resources WG will first establish a framework suited to the analysis and quantitation of potential issues, depending on the type and amount of input material, the assay(s) used, and the analysis method(s). Since the method used for the production of AIRR-seq data can impact the results, as shown by the benchmarking of TR library preparation methods study (Barennes et al., 2021), we plan to evaluate different standards in the framework of a molecular biology method benchmarking study as well. For the TR repertoire, we will take advantage of the already evaluated methods to include more gDNA-based methods. For the IG repertoire analysis, we will launch a systematic study, leveraging high-volume pooled-cell collections and synthetic standards that can be shared by investigators at multiple sites. We plan four major experiments: (1) analysis of TR and IG rearrangements in PBMCs using bulk gDNA and RNA approaches; (2) analysis of TR on sorted naïve polyclonal T cells using bulk gDNA; (3) analysis of IG rearrangements on sorted naïve polyclonal B cells using bulk gDNA and RNA; and (4) analysis of IG rearrangements on spleen cells from organ donors using bulk gDNA and RNA approaches. Spleen cells are enriched for memory B-cell clones and are useful for modeling clonal expansion and somatic hypermutation (Meng et al., 2017). Synthetic controls and UMIs (in the case of RNA-based sequencing) will be added to triplicate samples of PBMCs and polyclonal splenocytes. Samples will be run with and without spike-in controls that will be included at different ratios. Ideally, the selected methods will all be handle by 2 to 3 labs, a compromise between feasibility and inter-lab validation. Protocols and workflows will be standardized and shared. To avoid sequencing batch effect, we will sequence all the replicates through the same facility. Based on the results and to determine the lowest possible cell input levels, we will then evaluate the impact of decreasing the quantity of cells and repeat the same schema, focusing on the most reproducible methods. Finally, we will work closely with other AIRR Community working groups and additional experts in the field to harmonize standardization efforts. Together with the AIRR Community Software WG, we will select a series of tools for data quality control, alignment, and annotation and identify the analysis pipelines required for the detection of contamination, amplification bias, and batch effects. By leveraging the diverse skills of AIRR Community investigators, the development, optimization, and dissemination of biological standards for AIRR-seq data should progress quickly. Such ambitious project will require financial support in order to help volunteer labs to handle the experiments, already under discussion at the level of the AIRR-community.

Using, testing, and comparing these standards is but the first step. Beyond that is their adoption by the wider scientific community. For widespread adoption, commercial and other partnerships are essential for high-quality production and dissemination of the standards. In addition, to ensure broad distribution, controls should either be free of significant intellectual property restrictions or, if proprietary, be well-documented. For this, the Biological Resources WG will reach out to academic research groups (e.g., IMGT), non-profit organizations (e.g., the Global Alliance for Genomics and Health), governmental organizations (e.g., the US Department of Commerce’s National Institute of Standards and Technology), as well as commercial entities (e.g., the ATCC). With these efforts, we will be able to improve the rigor, robustness and interchangeability of AIRR-seq studies, and to increase their utility for downstream applications, including clinical diagnostics.
